# Perspective and Surprise in the Floating World

**DOI:** 10.3201/eid2206.AC2206

**Published:** 2016-06

**Authors:** Byron Breedlove, Jared Friedberg

**Affiliations:** Centers for Disease Control and Prevention, Atlanta, Georgia, USA

**Keywords:** art science connection, emerging infectious diseases, art and medicine, about the cover, infectious diseases, Katsushika Hokusai, perspective and surprise in the floating world, woodblock color print, Mt. Fuji, respiratory diseases, public health

**Figure:**
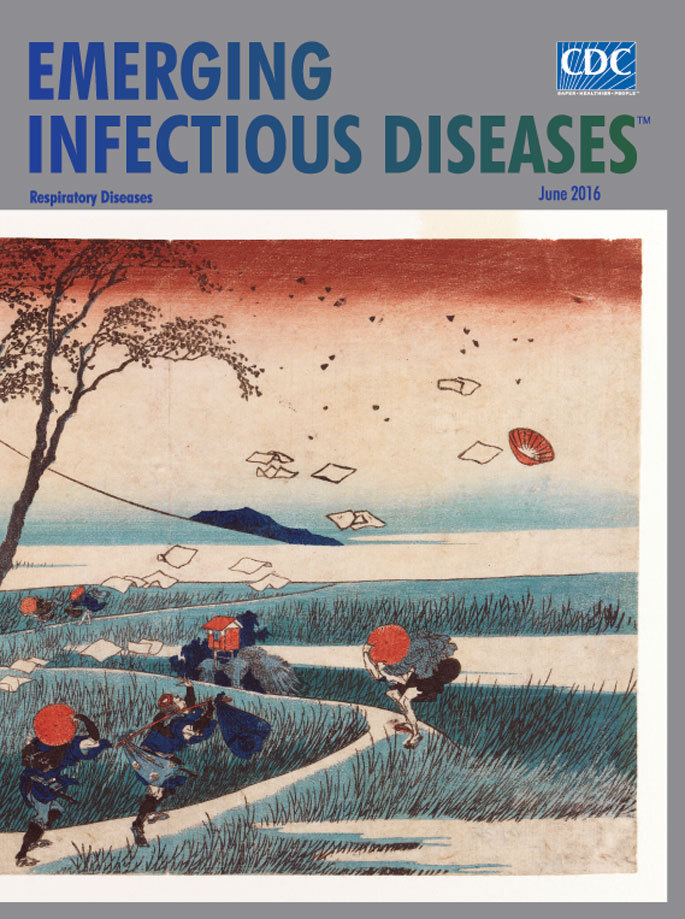
**Katsushika Hokusai (1760–1849). Yejiri Station, Province of Suruga, 1832. Woodblock color print, 9 9/16 in × 14 5/16 in/24.3 cm × 36.3 cm.** Brooklyn Museum, Brooklyn, NY; Gift of Frederic B. Pratt, 42.74

Katsushika Hokusai, one of the most prolific Japanese artists of the late Edo period (1615–1868), has been described as “cocky, quarrelsome, restless, aggressive, and sensational.” He obsessed with his art to the extent that he would move frequently rather than clean or repair his cluttered houses. During an artistic career that spanned 7 decades, he signed his works with more than 30 different names—typically in tandem with a shift in his artistic style and technique. Though this practice was common in Japan during his lifetime, Hokusai changed his name more often than any other major Japanese artist.

During his mid-70s, he used as his signature “Gakyo Rojin, Manji” or “The Art-Crazy Old Man.” His humorous, astute self-characterization is supported by the fact that he left more than 30,000 varied works of art, including woodblock prints, travel and book illustrations, manga, and silk paintings. Hokusai is one of the outstanding figures and innovators of the *Ukiyo-e* or “pictures of the floating world” (that is, scenes of everyday life) school of printmaking. He is credited with expanding the range of subjects from traditional *Ukiyo-e* depictions of courtesans and kabuki actors to encompass landscapes and to include people from various social levels.

Though the exact date of its publication is unknown, Hokusai’s defining work, “Thirty-Six Views of Mount Fuji,” was created from 1826 through 1833. Mt. Fuji, which is tied firmly into the cultural, spiritual, and social identity of Japan, had long served as inspiration for Hokusai and his art. In response to brisk sales of the initial set of 36, the artist expanded this popular collection to comprise 46 woodblock prints. Art scholar Sarah Thompson notes that the universal appeal of Hokusai’s works relates to his use of Western style perspectives and to “their strong internal structure. Many prints in the Fuji series explicitly contrast the strikingly triangular shape of the mountain with triangles, rectangles, trapezoids, or curved shapes formed by other elements of the design.”

Included among the original series of 36 views is this month’s cover image “Yejiri Station, Province of Suruga.” In this gently humorous image, Hokusai captures the reactions of several travelers on a serpentine path through the rustling marsh grasses, caught off guard by a sudden squall. Some stoop and clutch their hats and clothes as others fruitlessly reach toward their papers and hats blowing skyward, mixing with the leaves stripped from the bending trees. The British Museum offers this description: “The silhouette of Mt. Fuji is drawn with a single line, providing a backdrop for the figures and trees battling the wind in the foreground. Bending their bodies and clutching at scarves and hats, all turn their faces away from us—as if we were the source of the blast that carries off the tissues that had been tucked into the woman’s kimono.”

The element of surprise is central to Hokusai’s visual narrative. The Honolulu Museum of Art notes that Hokusai “focused on an instant of drama caused by a gust of wind. Fuji stands white and unshaken, affected neither by the wind nor the human drama.”

Like the unseen wind in Hokusai’s print, severe respiratory disease outbreaks can be sudden, disruptive, and chaotic. Emerging or reemerging pathogens may trigger serious, widespread respiratory disease outbreaks—including adenovirus bronchitis, influenza, Legionnaires’ disease, Middle East respiratory syndrome, multidrug-resistant tuberculosis, and severe acute respiratory syndrome (SARS).

To gain perspective on the nature of such threats, determining the etiology and understanding the clinical and epidemiologic characteristics of an outbreak is crucial. Surveillance systems—which are a basic component in the routine counting of disease cases—enable public health professionals to detect and investigate unexpected increases in these cases and prompt measures to control outbreaks, including those of emerging and reemerging respiratory disease. In a sense, surveillance systems embody the character of Mt. Fuji—which provides Hokusai’s travelers in “Yejiri Station, Province of Suruga” with a reference point during a storm—as they provide a constant, stable approach for outbreak investigations.
